# Supplementary material for: Individual differences in thinking style and dealing with contradiction: The mediating role of mixed emotions
Source: PLoS One. 2021 Sep 24;16(9):e0257864. doi: 10.1371/journal.pone.0257864 (PMC8462706; doi:10.1371/journal.pone.0257864)
Supplement: Attachment — Submitted filename: Cover letter mixed emotions & holism F.docx [file pone.0257864.s001.docx]

July 1^st^, 2021

Dr. Gaëtan Merlhiot,
Academic Editor
*PLOS ONE*

Dear Dr. Merlhiot,

We would like to express our sincere gratitude to you and the reviewers for the helpful and insightful feedback provided on June 4^th^ 2021 for our manuscript entitled “Individual differences in thinking style and dealing with contradiction: The mediating role of mixed emotions” (#PONE-D-21-07959). We were glad to see that our manuscript has merit.

Please, find below our responses to the concerns and comments raised by the reviewers. Please do not hesitate to contact us if you have any additional thoughts, questions, or queries concerning this revised manuscript. Thank you once more for inviting us to revise and resubmit our work. We believe that the revised manuscript is significantly improved and can make a more complete contribution to *PLOS ONE* than the previous version.

**Responses to Reviewer 1:**

Reviewer 1 provided a positive response to our manuscript and we very much appreciate it. This reviewer began by saying that our “article is well-written and meets most of PLOS ONE’s publication criteria” and that our “paper is strong—it has a plausible hypothesis that is connected to previous literature and testable with the present measures.” At the same time, Reviewer 1 raised a number of issues that we address below:

1. **Justification of the Locus of Attention subscale:** Reviewer 1 wondered why this subscale has been selected instead of the Attitudes Toward Contradiction subscale of the AHS.

We thank the reviewer for noticing this point and allowing us to clarify why this specific subscale has been chosen. In fact, the extent of specificity between the subscale of attitudes towards contradiction and the dependent variable (plausibility in contradictions) may be higher considering that this measure focuses on how people deal with two opposite propositions (an example item, “It is desirable to be in harmony, rather than in discord, with others of different opinions than one’s own”). However, we consider that this subscale is less suitable for capturing the variability of our proposed mediator (mixed emotions) insofar as it has nothing to do with how to deal with disagreements in points of view. That is why we chose a subscale with a broader focus that reflects the ability to maintain discrepant elements (whether emotions or opinions) simultaneously within the same reality.

According to the locus of attention subscale, holistic thinkers focus more on how the multiple components come together as part of a coherent overall experience compared to analytic processors who focus more on isolated components (an example item, “The whole, rather than its parts, should be considered in order to understand a phenomenon”). Although the specificity of this subscale might be lower with respect to the DV, we chose it because we consider that it is capable of encompassing mixed emotions and plausibility of the contradiction equally.

Furthermore, both scales keep a high correlation (*r =* .65; see Choi et al., 2007) as they are dimensions of the construct "Holistic-analytic thinking style" (regarding this last mention, we have expanded more information in the point #2, Reviewer 2). Someone might question whether it would not have been more appropriate to use the full scale with all its components, so this concern has been included in the part of limitations (page 23, lines 9-14 “Third, regarding the thinking style measure, instead of including the full Analysis-Holism Scale we used one of its subscales (i.e., locus of attention). Since the time and space to run the present studies were limited, we chose a costless measure without sacrificing reliability or validity. Future studies should also use a more complete measure of thinking style.”).

2. **Justification of the data analysis technique (SEM):** Reviewer 1 noted that the use of the technique (SEM) should be explained over the use of regression analysis.

We would like to thank the reviewer for this comment. In contrast with classic linear models (e.g., ANOVA, linear regression, generalized linear regression), SEM models allow researchers to control the measurement error, something crucial when dealing with latent variables, as it is the case of the holistic-analytic thinking style continuum.

We agree that a better justification for the analytical approach could be included in the manuscript. We have added a brief description of this approach in the statistical analyses section of the first study (page 11, paragraph 2).

3. **Inclusion of descriptive statistics and correlations:** Reviewer 1 asked us to include correlations and descriptive statistics of the measures.

We appreciate the comment and the possibility of including more information regarding our measures. We have added a brief descriptive analysis of the variables included in each study (see page 12, and pages 18-19), including the correlations between the statements in the first study, and between the statements and mixed emotions in the second.

3. **a. Measurement of the variable Plausibility of Contradictions:** Reviewer 1 wondered why we decided to dichotomize the criterion variable instead of using a continuous approach (e.g., summing up the two statements).

We thank the reviewer for the opportunity to explain our instantiation of the criterion variable. Taking the variable as a composite measure of the statements (i.e., summing or averaging the two statements) might lead to some problems with the conceptualization of the plausibility of contradictions. Please allow us to briefly explain our rationale. Imagine these 2 participants: Participant A considers the plausibility of both statements to be a 5 (out of 7), whereas participant B considered the plausibility of the first statement to be a 7 and the second statement to be a 3. Once we compute the composite index for each participant, both participants A and B receive the same score (i.e., 10). However, in this particular case, participant A showed a response pattern more consistent with the plausibility of contradictory information than participant B. Thus, we think that the continuous method of scoring the DV does not distinguish between participants in terms of how much they compromise when dealing with contradictory information, at least when it comes to participants who provide responses around the mid-point.

We decided, however, to report this other way of computing the criterion variable using the sum of the two statements as an overall indicator of plausibility in contradictory information (see page 14, paragraph 2, and page 20, paragraph 2), such that higher scores indicated that the two statements were perceived as highly plausible and lower scores indicated that one of the statements was perceived as less plausible that the other. When using this way of scoring the criterion variable, the data fitted in Study 1, χ2 (13) = 35.81, CFI = .97, TLI = .95, RMSEA (95% CI) = .059 (.041, .077), and Study 2, χ2 (18) = 74.54, CFI = .95, TLI = .92, RMSEA (95% CI) = .071 (.057, .087). This analyzes were already included in the previous version of the manuscript.

3. **b. Different analytical approach:** Reviewer 1 suggested to model whether thinking style moderates the relation between the two plausibility statements.

In point #2 above, we have explained why we have chosen a SEM approach rather than treating the variables as observed and using a regression approach. Nonetheless, we have modeled our results by using the approach the reviewer proposed and the results are consistent with our findings. In this regression approach, we used each statement separately and continuously. Thus, Statement 1, Thinking Style (the mean of the 6 items), and the interaction term (i.e., Statement 1 × Thinking Style) were used as predictors, and Statement 2 was used as the criterion variable. The criterion variable (Statement 2) was submitted to a multiple regression analysis. The key two-way interaction was computed using the PROCESS macro for SPSS (model 1; Hayes, 2013). The continuous variables (i.e., Statement 1 and Thinking Style) were mean-centered to reduce multi-collinearity issues when computing interaction terms. The criterion variable (i.e., Statement 2) was regressed onto the predictors (Statement 1 and Thinking Style) as well as their interaction term using a hierarchical regression (i.e., main effects in the first step, followed by two-way interaction). Based on Cohen and Cohen (1983), all main effects and interactions were interpreted in the first block in which they are shown in the regression analyses.

The regression analysis revealed a main effect of statement 1 on statement 2, *B* = -0.280, *t*(682) = -6.751, *p* < .001, indicating that the more plausible individuals perceive statement 1, the less plausible they perceive statement 2 and vice versa.

Interestingly, this main effect was qualified by a significant interaction between statement 1 and thinking style, *B* = 0.074, *t*(681) = 2.047, *p* = .041. As illustrated in Figure 1, among those with lower AHS scores (-1SD; people who tend to be more analytic), statement 1 was negatively related to statement 2, *B* = -0.347, *t*(681) = -6.573, *p* < .001. For those with higher AHS scores (+1SD; people who tend to be more holistic), statement 1 was also negatively related to statement 2, *B* = -0.201, *t*(681) = -3.552, *p* = .0004, but this negative correlation was significantly less stronger as indicated by the significant two-way interaction (thus indicating greater plausibility in contradiction).

This regression approach yielded very similar results compared to our SEM approach. That is, a higher plausibility of contradiction among individuals who tend to be more (vs. less) holistic.

In Study 2, the findings were less robust although the pattern of results was replicated. The relationship between Statement 1 and Statement 2 as a function of Thinking Style approached significance, *B* = 0.063, *t*(620) = 1.775, *p* = .076.

Figure 1. Relationship between Statement 1 and Statement 2 as a function of Thinking Style.

We provided these analyses to show the reviewer that both procedures lead to similar conclusions. Although we agree with the reviewer that modeling the interaction between the statements could be interesting, we do not deem necessary to add it to the manuscript as it is somewhat redundant with the analyses currently reported in the paper.

In addition, modelling this type of moderations in SEM is tricky, as one of the predictors is a latent variable (Thinking Style) and the other predictor is an observed variable of just one item (Statement 1). Many authors have argued that this moderation in SEM can produce problems of convergence of the model (Foldnes & Hagtvet, 2014). However, we still think that our SEM approach provides more information than the regression approach for the reasons explained in point #2.

Cohen J, Cohen P, West SG, Aiken LS. Applied multiple regression. Correlation Analysis for the Behavioral Sciences. 1983;2.

Foldnes N, Hagtvet KA. The choice of product indicators in latent variable interaction models: Post hoc analyses. Psychol Methods. 2014 Sep;19(3):444-57.

Hayes AF. Introduction to mediation, moderation, and conditional process analysis: A regression-based approach. Guilford publications; 2017 Dec 13.

3. **c. Measurement of the variable Mixed Emotions:** Reviewer 1 was concerned about the measurement of mixed emotions and that we did not take into account the intensity of the mixed state. Also, this reviewer asked us to include descriptive data or a graph showing where the data are concentrated.

This is a fair comment. The research question proposed in this work is whether the occurrence of mixed emotions can serve as a mediator mechanism between thinking style and plausibility in contradictions regardless of the intensity of the emotions. To test this hypothesis, we used an operationalization of mixed emotions following previous research (Hamamura et al., 2008; Hui et al., 2009), and as the reviewer points out, this does not capture the intensity of the emotions experienced. Given that it seems to us an interesting question, we have included in the discussion the proposal of considering the role of the intensity of mixed emotions in subsequent studies (see limitations and future research part, page 23, lines 15-19). Furthermore, since we do not want to mislead the reader on this aspect, we have deleted the mention of intensity in the method section (in such a way that in the new manuscript it appears only in the discussion). We thank Reviewer 1 for this suggestion that raises interesting lines of follow-up research to this one.

[…] Forth, regarding the variable mixed emotions, someone might wonder what is the role of the intensity of mixed emotions. In this research, we have focused on the occurrence of mixed emotions but not on the degree to which people experience this emotional state. This is an interesting question that is worth exploring in further studies, either by manipulating or measuring experiences of mixed emotions and their intensity. […]

4. **Sample size:** Reviewer 1 asked us what strategy we used to determine the sample size.

Sample size was determined following the recommendations of several papers on the topic. In SEM, the statistical power depends on model complexity, the estimation method, and the response format of the observed variables (i.e., items). Hence, there are no clear rules of thumb to determine the minimum sample size. For a 3-factor correlated model with categorical data, a sample size of 400-500 participants is needed to ensure acceptable Type I and Type II error rates (Bandalos, 2014; Forero et al., 2009; Wolf et al, 2013). In general, when using a SEM approach, the higher the sample size, the better.

The sample size of both studies is above these minimum requirement of participants to ensure enough statistical power.

Bandalos DL. Relative performance of categorical diagonally weighted least squares and robust maximum likelihood estimation. Struct Equ Modeling. 2014;21(1):102–16.

Forero CG, Maydeu-Olivares A, Gallardo-Pujol D. Factor analysis with ordinal indicators: A Monte Carlo study comparing DWLS and ULS estimation. Struct Equ Modeling. 2009;16(4):625–41.

Wolf EJ, Harrington KM, Clark SL, Miller MW. Sample size requirements for structural equation models: An evaluation of power, bias, and solution propriety: An evaluation of power, bias, and solution propriety. Educ Psychol Meas. 2013;76(6):913–34.

5. **Open data:** Reviewer 1 could not access the data on OSF.

Thank you for noticing this issue. We have granted access to the link to anyone but anonymized for blind review purposes. The new link that we have included in the manuscript is: <https://osf.io/yadft/?view_only=f1f8cefed3f144a9bd7f8d3a888dc476>

**Responses to Reviewer 2:**

Reviewer 2 expressed that “I found this study truly interesting.” We really appreciate this positive comment and the feedback of their review. Furthermore, this Reviewer brought up additional comments that we address below:

1. **Measurement of Holistic-Analytic Thinking Style:** Reviewer 2 expressed that there were other procedures to measure holistic-analytic thinking style that could have used. At the same time, this reviewer was concerned about the validity of the scale we used to measure thinking styles.

In the present research, we have opted to measure the key construct by using a self-reported measure (i.e., the Locus of Attention subscale from the AHS). As the reviewer points out, there exist multiple procedures to measure holistic-analytic thinking (for a review, see Na et al., 2020). The original work by Choi et al. (2007) demonstrates that the AHS is both a reliable and valid instrument to measure systematic cognitive differences in holistic-analytic thinking. Specifically, this measure has been shown to predict performance on two cognitive tasks (categorization and causal reasoning). Nonetheless, we now acknowledge in the general discussion that future research could not only manipulate thinking styles but also measure them with different procedures, such as the framed line test or the triad task (for a review, see Na et al., 2020).

Na J, Grossmann I, Varnum ME, Karasawa M, Cho Y, Kitayama S, Nisbett RE. Culture and personality revisited: Behavioral profiles and within‐person stability in interdependent (vs. independent) social orientation and holistic (vs. analytic) cognitive style. J of Pers. 2020 Oct;88(5):908-24.

2. **Overarching definition of holistic thinking:** Reviewer 2 asked us to extend our conceptualization of holistic-analytic thinking style.

We have followed this recommendation and the revised manuscript now includes a paragraph that addresses the different components that define holistic-analytic thinking style. These new lines reflect the multifaceted nature of the construct by explicitly mentioning and describing its dimensions (page 3, second paragraph).

[…] These theories define holistic-analytic thinking style as a multifaceted construct composed by several dimensions. The first dimension is *causality*, which considers the presence of complex causalities and the elements of the universe as interconnected and interrelated (vs. the universe consists of elements that are independent of each other, Koo & Choi, 2005). The second one is *attitudes towards contradiction*, which assesses the preference for resolving the contradiction through a reconciliation strategy, seeking the “middle way” between opposing propositions (vs. contradictions are resolved by choosing one of the two opposite propositions, Spencer-Rodgers et al., 2010). The third, *perception of change*, refers to the tendency to perceive the elements as being in constant change and unpredictable (vs. linear changes and predictable, Yama & Zakaria, 2019). The last dimension is *locus of attention*, which places the focus on “the big picture”, considering the elements of the stimulus as a whole (rather than decomposing the stimulus in their parts ignoring the context, Nisbett & Miyamoto, 2005). […]

Koo M, Choi I. Becoming a holistic thinker: training effect of oriental medicine on reasoning. Pers Soc Psychol Bull. 2005;31(9):1264–72.

Nisbett RE, Miyamoto Y. The influence of culture: holistic versus analytic perception. Trends Cogn Sci. 2005;9(10):467–73.

Spencer-Rodgers J, Williams MJ, Kaiping Peng. Cultural differences in expectations of change and tolerance for contradiction: a decade of empirical research. Pers Soc Psychol Rev. 2010;14(3):296–312.

Yama H, Zakaria N. Explanations for cultural differences in thinking: Easterners’ dialectical thinking and Westerners’ linear thinking. J Cogn Psychol (Hove). 2019;31(4):487–506.

3. **Mediation model:** Reviewer 2 was concerned about the reverse-causality problem in correlational designs. Also, this reviewer questioned wording indicating a more causal role of the variables.

We agree with Reviewer 2 about the correlational nature of our mediation. We also agree that the causality implied by some sentences is not warranted by the design of our study. Therefore, we now use more accurate wording to express the correlational nature of this mediation model throughout the manuscript.

Finally, in addition to the changes in wording, we have considered important to clarify further this issue in the limitations section of the manuscript: “Finally, we acknowledge that a design with an experimental approach would provide evidence for causal mediation [71, 72]. In fact, the correlational nature of our design prevents us to conclude whether there is a model with more explanatory power than the one proposed.” (page 23, lines 20-23).

71. Fiedler K, Harris C, Schott M. Unwarranted inferences from statistical mediation tests–An analysis of articles published in 2015. J Exp Soc Psychol. 2018 Mar 1;75:95-102.

72. Lemmer G, Gollwitzer M. The “true” indirect effect won't (always) stand up: When and why reverse mediation testing fails. J Exp Soc Psychol. 2017 Mar 1;69:144-9.

4. **Sample size:** Similarly to point **#**4 of Reviewer 1, this Reviewer also asked us what strategy we used to determine the sample size and whether there was a stopping rule.

We aimed for a sample size above 500 participants (see response to point #4 of Reviewer 1). Given that the on-line sampling strategy have some tradeoffs (is cost-efficient, but it is necessary to control the quality of the answers given by the participants), we decided to stop the data collection when the sample size approached N = 650. We opted for this number in case some participants failed the attention checks of the study and need to be removed from the study (something that only could be assessed after the data collection).

**Summary**

We hope that this letter clarifies how we have addressed the key issues raised during the review process. Some of the issues were addressed by attempting to clarify our writing, and others were addressed with new analysis of the data. The feedback from you and both reviewers has certainly helped us to improve the manuscript quality considerably. Because the reviewers seemed positively disposed toward the potential contribution of the paper in their reviews, as did you, we hope that you find this version –which addresses the concerns mentioned during the review process - suitable for publication. Please do not hesitate to contact us if you have any additional query.

Best regards,

The authors.
