# Supplementary material for: Individual differences in thinking style and dealing with contradiction: The mediating role of mixed emotions
Source: PLoS One. 2021 Sep 24;16(9):e0257864. doi: 10.1371/journal.pone.0257864 (PMC8462706; doi:10.1371/journal.pone.0257864)
Supplement: Attachment — Submitted filename: Cover letter Plos One R2.docx [file pone.0257864.s002.docx]

September 6^th^, 2021

Dr. Gaëtan Merlhiot,
Academic Editor
*PLOS ONE*

Dear Dr. Merlhiot,

We are very thankful for the opportunity to resubmit our work entitled “Individual differences in thinking style and dealing with contradiction: The mediating role of mixed emotions” (#PONE-D-21-07959) to *PLOS ONE*. We are delighted to see that we have successfully addressed the comments and concerns of the reviewers in the first round and that they were very enthusiastic about the contribution of our work.

In this second round, we have addressed the a suggestion raised by the Reviewer 1.

**Response to Reviewer 1:**

Reviewer 1 noted that we have addressed all of his/her concerns and considers this a good paper with potential contribution to the literature. We are really happy to hear these encouraging comments.

**Response to Reviewer 2:**

We would like to thank Reviewer 2 for his appreciation of our changes in the revised version of the manuscript. He (self-identified reviewer) only raised a minor issue regarding the appropriate use of variable labels according to a correlational design. We agree that the correct use of the labels should be Predictor and Outcome instead of Independent and Dependent Variables. Therefore, we have modified the terms to conform to the language of correlational designs throughout the manuscript. We thank Onurcan Yilmaz for his helpful feedback along the process.

**Summary**

We hope this letter clarifies how we have addressed the few remaining issues raised during this second review round. Furthermore, we would like to sincerely thank reviewers for their time and constructive feedback during the entire review process which has helped us to considerably improve the paper. Please do not hesitate to contact us if you have any additional query.

Best regards,

The authors
